# The *amyR*-deletion strain of *Aspergillus niger* CICC2462 is a suitable host strain to express secreted protein with a low background

**DOI:** 10.1186/s12934-016-0463-1

**Published:** 2016-04-28

**Authors:** Hui Zhang, Shuang Wang, Xiang xiang Zhang, Wei Ji, Fuping Song, Yue Zhao, Jie Li

**Affiliations:** Northeast Agricultural University College of Life Science, Harbin, 150030 China; State Key Laboratory for Biology of Plant Diseases and Insect Pests, Institute of Plant Protection, Chinese Academy of Agricultural Sciences, Beijing, 100193 China

**Keywords:** *Aspergillus niger*, *amyR*, Regulation, Proteomics, Transcriptome

## Abstract

**Background:**

The filamentous fungus *Aspergillus niger* is widely exploited as an important expression host for industrial production. The glucoamylase high-producing strain *A. niger* CICC2462 has been used as a host strain for the establishment of a secretion expression system. It expresses recombinant xylanase, mannase and asparaginase at a high level, but some high secretory background proteins in these recombinant strains still remain, such as alpha-amylase and alpha-glucosidase; lead to a low-purity of fermentation products. The aim was to construct an *A. niger* host strain with a low background of protein secretion.

**Results:**

The transcription factor *amyR* was deleted in *A. niger* CICC2462, and the results from enzyme activity assays and SDS-PAGE analysis showed that the glucoamylase and amylase activities of the ∆*amyR* strains were significantly lower than those of the wild-type strain. High-throughput RNA-sequencing and shotgun LC–MS/MS proteomic technology analysis demonstrated that the expression of amylolytic enzymes was decreased at both the transcriptional and translational levels in the ∆*amyR* strain. Interestingly, the ∆*amyR* strain growth rate better than the wild-type strain.

**Conclusions:**

Our findings clearly indicated that the ∆*amyR* strain of *A. niger* CICC2462 can be used as a host strain with a low background of protein secretion.

## Background

The filamentous fungus *Aspergillus niger* is one of the most important industrial filamentous species and is used extensively for the production of organic acids and industrial enzymes [1–3] and for basic genetic research. Compared with *E. coli* and *Pichia* expression systems, *Aspergillus* has better expression and secretion capacity [4, 5]. The GRAS (generally recognized as safe) status of *A. niger* makes it attractive as a host for recombinant protein expression, and *A. niger* is known as “a cell factory of eukaryotic protein expression [6, 7]”. The main challenge in industry using filamentous fungi is the expression of homologous and/or heterologous proteins that are functional. Strategies for improving protein production have been discussed in detail by Archer et al. in 1994 [8], including the use of strong homologous promoters, increased gene copy number and gene fusions etc. Nevalainen and Peterson [9] described the current obstacles for the production of recombinant proteins, including the mode of glycosylation and the problems related to the processing in the endoplasmic reticulum. They also proposed that exploration of metabolic pathway engineering may result in the improvement of the production of recombinant proteins.

The glucoamylase-producing strain *A. niger* CICC2462 has been used as a host strain for the establishment of a secretion expression system by our research team. The target gene was integrated into the *glaA* gene locus for high expression by homologous gene replacement. It could express recombinant xylanase, mannase and asparaginase at a high level [10–12], but some high secretory background proteins still remain, such as alpha-amylase and alpha-glucosidase, the low proportion of target proteins relative to the total protein not only restrict the continued ascension of target protein production but also lead to a low-purity of fermentation products, thus increasing the costs of target protein purification. Therefore, one possible method that could be effective to solve this problem is to regulate highly expressed genes at the transcriptional level and subsequently reduce the amount of secretory proteins in the whole expression system.

The regulation of secretory proteins in *Aspergillus* species has been well studied. The *cis*-elements SYGGRG, CGGN_8_(C/A)GG, GGCTAR and CCAAT have been found upstream of the major secretory protein genes amylase, cellulase and hemicellulase, which are regulated by the regulatory factors CreA/B/C, AmyR, XlnR, ClbR and the Hap complex [13, 14]. AmyR is a Zn(II)_2_Cys_6_-type transcriptional activator responsible for the induction of the amylolytic genes in *Aspergillus* species, and is known to bind to the CGGN_8_(C/A)GG sequence in various amylase promoters to activate gene transcription [15–17]. Furthermore, many details of the structure and regulatory function of the *amyR* gene have been elucidated [18–20].

In the post-genomics era, various-omics technologies have been applied in filamentous fungus to generate a new approach for improving the expression system of host strains for the industrial production of proteins. Proteomic analysis is a powerful tool for high-throughput global protein expression analysis using gel-based or gel-free protein separation techniques coupled with mass spectrometry (MS/MS). Proteomic methods have been used to study the effect of different culture conditions on the secretome of *A. niger* [21–23]. Furthermore, recent advances in high-throughput RNA sequencing (RNA-Seq) technology have markedly reshaped the landscape of transcriptome analysis [24, 25]. Transcriptomics sequences of *A. niger* strains have been used to give new knowledge about the regulation of carbohydrate metabolism [26, 27]. Such knowledge could provide new strategies for strain improvement.

In this study, an *amyR*-deletion strain was constructed, transcriptome, secretome, amylolytic enzyme activity and growth rate were analysed and compared in the amyR deletion strain and the wild-type strain. Ultimately, a host strain with a low background of protein secretion and without growth inhibition was obtained.

## Methods

### Strains and culture conditions

*Aspergillus niger* CICC2462 was provided by an enzyme preparation company (Zhaodong Richeng Enzyme Preparation Co., Ltd.), and the pSZH-xynB plasmid vector was constructed by our laboratory. *Escherichia coli* DH5α, *Agrobacteriumtumefaciens* AGLI and the pAN7-1 vector were used for DNA manipulation. A mutant strain (∆*amyR*) of *A. niger* CICC2462 was constructed in this study. The *A. niger* strain was grown at 30 °C in PDA medium (20 g/L glucose, 3 g/L KH_2_PO_4_, 1.5 g/L MgSO_4_·7H_2_O, and 200 g/L potato piece). Plasmid-harbouring *E. coli* cells were grown at 37 °C in LB medium (5 g/L yeast extract, 10 g/L peptone, and 10 g/L NaCl, pH 7.0). *Agrobacteriumtumefaciens* was grown at 28 °C in YEB medium (1 g/L yeast extract, 5 g/L peptone, 0.493 g/L MgSO_4_·7H_2_O, 5 g/L beef extract paste, and 5 g/L sucrose, pH 6.5). Cultures where grown in shake flasks at 30 °C in industrial fermentation medium (100 g/L glucose, 20 mL/L corn steep liquor, and 20 g/L soybean powder, pH 5.5–6.0) at 260 rpm/min with 10 % of the inoculation amount.

### Shotgun LC–MS/MS proteome analysis

Shotgun LC–MS/MS was performed at Shanghai GeneCore Bio-Technologies Co., Ltd. (Shanghai, China). Peptides were purified through reverse-phase high-performance liquid chromatography on a surveyor LC system (Thermo Finnigan, San Jose, CA, USA) with an autosampler. Peptides were ionized in the positive ion mode and introduced into an LTQ linear ion trap mass spectrometer equipped with a microelectrospray source for MS/MS. Protein identification was performed with Bioworks Browser 3.3 against the NCBI Uniprot *A. niger* database and the results were extracted from the SEQUEST file using in-house software. The SEQUEST search parameters were Delta CN (≥0.1) and Xcorr (one charge ≥1.9, two charges ≥2.2, three charges ≥3.75). Proteins with a unique peptide count ≥2 were considered accurately identified and used for bioinformatic analysis.

### Construction of *amyR*-deletion cassettes

Plasmid vectors were constructed using a previously published enzymatic assembly method [28]. The PCR primers are listed in Table 1. The *amyR* gene deletion vector, pAmyRd, was constructed in three steps as follows. First, a 1.3-kb *Spe*I/*Hind*III amyR3SH fragment was amplified from *A. niger* CICC2462 genomic DNA with the primer pairs amyR3SH-R/amyR3SH-F, and the amyR3SH fragment was placed between the *Xba*I and *Hind*III sites located immediately in front of the *hph* gene on pAN7-1 to construct pAN-amyR3SH. Second, this plasmid was introduced between the *Xho*I and *Hind*III sites of pSZH-xynB to yield the plasmid pSZH-amyR3SH. Finally, a 1.2-kb *Xba*I/*Kpn*I amyR5 fragment and a 1.3-kb *Xho*I/*Kpn*I amyR3KX fragment were amplified from *A. niger* CICC2462 genomic DNA with the primer pairs amyR5-R/amyR5-F and amyR3KX-R/amyR3KX-F, respectively. These fragments were then introduced between the *Xba*I and *Xho*I sites of pSZH-amyR3SH to obtain *amyR* gene-deletion vector, pAmyRd. This vector was subsequently used for the transformation of *A. niger* CICC2462 to obtain the reporter *A. niger* ∆*amyR* strain.

**Table 1:** PCR primers used in this study

Primers	Sequence (5′–3′)	Enzyme sits
amyR3SH	ACTAGTGCCCCCTACATCTGCTATT	*Spe*I
	AAGCTTCCACCACCATCAAAATCAC	*Hind*III
amyR3KX	GGTACCTGCCCCCTACATCTGCTATT	*Kpn*I
	CTCGAGTTCCACCACCATCAAAATCAC	*Xho*I
amyR5	TCTAGACCAGATGTGCTCAAGGAATG	*Xba*I
	GGTACCAGAAGCGTAGGCTGAACCAT	*Kpn*I
glaA	GACAATGGCTACACCAGCAC	
	AATCACACCAGGAGCAGGAC	
amyR	CGAGCCGTTCTCTCAGTTTC	
	TGGTGGCAATCTTGTTGAAG	
amyA	GCCCATCTACAAAGACGACA	
	ACATTTCCATCCGAACCAAC	
actA	CCACGAGACCACCTTCAACTCCA	
	CCACCGATCCAGACGGAGTACTTGC	
creA	CGCAATCACCATTTGTTCAG	
	TGGGAGAGGAAGGAGCAGT	

### *Agrobacterium tumefaciens*-mediated transformation of *Aspergillus niger*

The following steps were taking throughout the procedure: a pAmyRd *Agrobacterium* transformant colony which was obtained by the freeze-thawing method in YEB liquid medium (containing 50 mg/L rifampicin and 100 mg/L kanamycin) and incubated at 28 °C with 200 rpm rotary shaking for 24 h. The pAmyRd *Agrobacterium* was inoculated at a proportion of 1:10 into fresh YEB liquid medium (containing 50 mg/L rifampicin and 100 mg/L kanamycin) for secondary activation until the *OD*_600_ reached 0.4–0.6. 80 μL of the *A. niger* CICC2462 mycelium suspension and 80 μL of the pAmyRd *Agrobacterium* suspension were coated on a covering cellophane PDA solid medium (containing 200 μmol/L acetosyringone). The cellophane was transferred to a new PDA solid medium (containing 200 mmol/L cefotaxime sodium and 200 mmol/L hygromycin B) after being co-cultivated at 28 °C for 2 days and removed it after cultivation at 30 °C for 1 day. Cultivation for 6–8 days until growth of the *A.niger* colonies were detected. The resistant *A.niger* colony was transfered to PDA liquid medium (containing 200 mmol/L hygromycin B) for secondary screening. *A. niger* genomic DNA was extracted for identification. The primer pairs amyR5-R and amyR3KX-F were used for PCR identification.

### Enzyme and protein assays

Glucoamylase activity was assayed using the DNS method. Protein fermentation supernatant samples were mixed with an equal volume of 2× protein loading buffer and boiled for 10 min. The samples were subjected to SDS-PAGE using 4 % stacking gels and 12 % resolving gels in a mini-vertical electrophoresis system (Bio-Rad Laboratories, USA). The gels were stained with CBB-R250.

### RNA isolation, cDNA synthesis and quantitative RT-PCR

Total RNA extraction, cDNA synthesis and quantitative real-time PCR were performed. An RNA isolation reagent was used for the total RNA extraction, and the PrimeScript RT Reagent Kit with gDNA Eraser was used for cDNA synthesis according to the manufacturer’s protocols. Quantitative real-time PCR was performed on a Stratagene Mx3000P instrument (USA) using SYBR Premix Ex Taq. Quantitative RT-PCR analysis of each gene was performed in triplicate with the primers listed in Table 1. Each amplification reaction was conducted in a total reaction volume of 20 μL. The thermal cycling protocol was as follows: initial denaturation for 2 min at 95 °C followed by 40 cycles of 10 s at 95 °C and 30 s at 60 °C. The fluorescence signal was measured at the end of each extension step at 95 °C. The transcript number of the actin gene from the same sample was used as an internal quantitative standard.

### Transcriptome sequencing and annotation

Transcriptome sequencing of the *A. niger* CICC2462 and ∆*amyR* strains were performed with an Illumina HiSeq™2000 system. Reads were mapped to the reference genome of *A. niger* CBS513.88. The possible functions of all unigenes and the differentially expressed unigenes were determined with the Gene Ontology (GO) classification system [29]. The Gene Ontology terms for each *A. niger* unigene were obtained with the software Blast2GO using the default parameters. GO enrichment analyses were performed with the DAVID database. Blast2GO was also used for the GO functional enrichment analysis of certain genes by performing Fisher’s exact test with a robust false discovery rate (FDR) correction to obtain an adjusted *P* value between the gene and the entire annotation.

## Results

### *amyR* gene deletion decreases the amount of secretory proteins in *A. niger* CICC2462

Previous studies have shown that amylolytic-related proteins are regulated by the *amyR* transcriptional factor [15, 16]. To decrease the total quantity of proteins secreted in *A. niger* CICC2462, a deletion vector (pAmyRd) was transformed into it using the *Agrobacterium tumefaciens*-mediated method. Through PCR analysis, we found that 11 out of 14 transformants were positive recombinant transformants (primers of amyR5-R and amyR3KX-F), resulting in a transformation efficiency of 78.5 %, and three transformants of the ∆*amyR* strain (amyR3, amyR6, and amyR12) were further characterized.

**Fig. 1:**
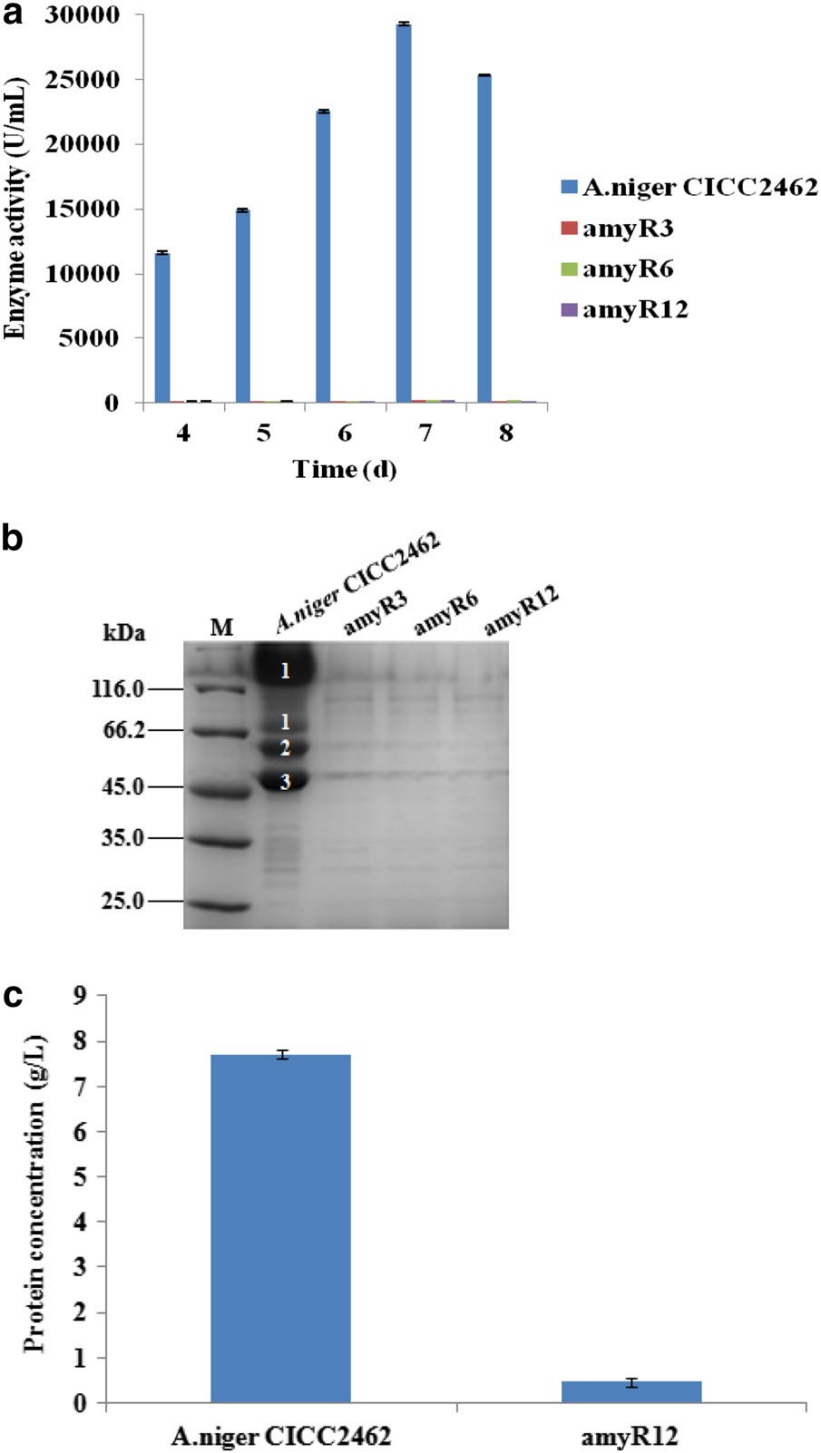
**a** Activities of amylolytic enzymes from *A. niger* CICC2462 and ∆*amyR* (amyR3, amyR6 and amyR12) strains grown on fermentation medium. **b** Separation of identified amylolytic enzyme proteins from the fermentation supernatant of *A. niger* CICC2462 and ∆*amyR* (amyR3, amyR6 and amyR12) strains by SDS-PAGE. 1, 2 and 3 represent glucoamylase, acid-stable alpha-amylase and alpha-amylase, respectively. An volume of 10 μL of each sample was separated on a 12 % resolving gel. **c** The protein concentration of *A. niger* CICC2462 and amyR12 strains

The enzyme activities of the fermentation supernatant of the reference strain *A. niger* CICC2462 and the ∆*amyR* strain were measured. The enzyme activities reached a peak after 7 days of culture. The data showed that the amylolytic enzyme activities of the ∆*amyR* strain was barely detected and they were 144- to 157-fold lower than that of the *A. niger* CICC2462 strain (Fig. 1a). SDS-PAGE analysis demonstrated that the protein bands corresponding to glucoamylase, acid-stable amylase A and alpha-amylase A were absent in the ∆*amyR* strains (Fig. 1b). These results suggest that the expression of *glaA*, *asAA* and *amyA* in *A. niger* CICC2462 depends on the presence of the *amyR* gene. The amount of total protein was assayed and compared in the supernatant after growth of the two strains through a BCA protein assay kit. The protein concentration of *A.niger* CICC2462 and amyR12 strain was 7.70 and 0.47 g/L, respectively. The date showed that the ∆*amyR* strain was 16.4-fold lower than that of the *A.niger* CICC2462 strain (Fig. 1c).

Furthermore, samples from the fermentation supernatant used for enzyme activity analysis were also analyzed at the proteomic level using shotgun LC–MS/MS directly. The main metabolic enzymes are listed in Tables 2 and 3. By comparing the major identified proteins with the *A. niger* CICC2462 proteomics, as observed from the number of peptides from the two strains, the overall quantity of proteins decreased. The proteins presenting the greatest reductions were identified as glucoamylase and amylase. Proteases also decreased, whereas alpha-galactosidase B and beta-mannosidase A increased, xylanase presented both decreased and increased.

**Table 2:** Top 15 most abundant proteins of *A. niger* CICC2462 identified by shotgun LC–MS/MS

No.	Protein description	GN	Pep count^a^	Unique pepcount^b^	Cover percent (%)^c^
1	Glucoamylase	glaA	1022	19	59.22
2	Acid-stable alpha-amylase (fragment)	amyA	358	15	53.51
3	Extracellular alpha-amylase amyA/amyB	amyA	286	16	41.77
4	Acid alpha-amylase	amyA	236	10	38.22
5	Putative uncharacterized protein An01g10930	An01g10930	85	15	27.98
6	Hydrolysis of the 1	An02g07020	39	8	40.00
7	Probable alpha-galactosidase B	aglB	34	7	25.28
8	Catalases convert 2 H(2)O(2) to O(2) + 2 H(2)O	An01g01550	30	12	26.13
9	Endoglucanase A	eglA	29	5	27.62
10	Aspartic proteinase	An01g00370	28	11	42.49
11	Probable beta-mannosidase A	mndA	26	13	23.85
12	Similarity to isoamyl alcohol oxidase mreA	An03g06270	26	9	24.78
13	Hypothetical protein	An01g14730	23	9	47.06
14	Probable endo-1,4-beta-xylanase C	xlnC	20	7	36.39
15	Probable glucan endo-1,3-beta-glucosidase	eglC	14	4	17.17

^a^The pepcount refers to the number of total peptides assigned to proteins

^b^The unique pepcount refers to the number of different peptides assigned to the proteins

^c^The percentage coverage is defined as the ratio (%) of the protein sequence covered by the matched peptides

**Table 3:** The major proteins of *amyR12* identified by shotgun LC–MS/MS

Protein description	GN	Pep count^a^	Unique pepcount^b^	Cover percent (%)^c^
Glucoamylase	glaA	66	13	38.91
Alpha-amylase A	amyA	61	11	26.65
Probable alpha-galactosidase B	aglB	111	10	27.54
Endoglucanase A	eglA	26	6	26.47
Probable glucan endo-1,3-beta-glucosidase	eglC	19	5	11.96
Aspartic proteinase	pep1	11	5	11.25
Beta-mannosidase A	mndA	101	22	28.03
Mannan endo-1,4-beta-mannosidase	man26A	5	4	13.73
Endo-1,4-beta-xylanase	xynA	2	2	8.23
Endo-1,4-beta-xylanase (fragment)	xynV	32	2	12.76
Beta-xylanase		18	12	35.78

^a^The pepcount refers to the number of total peptides assigned to proteins

^b^The unique pepcount refers to the number of different peptides assigned to the proteins

^c^The percentage coverage is defined as the ratio (%) of the protein sequence covered by the matched peptides

### Transcriptome analysis of *A. niger* strains

#### RNA-Seq data analysis

A total of 49,072,138 and 48,989,116 high-quality reads were obtained from two strains (*A. niger* CICC2462 and amyR12) by RNA-Seq, respectively. We specifically observed 11,699 and 11,646 expressed genes from these two strains (see Table 4). A total of 1956 differentially expressed unigenes (DEUs) between *A. niger* CICC2462 and amyR12 were detected, and these included 1331 and 635 unigenes that were up- and down-regulated in amyR12, respectively. Of the total reads, 89.19 and 87.93 % matched to unique genomic locations, respectively, whereas 54.31 and 53.36 % matched to unique gene locations, respectively. To better analyse the genes that presented marked differences, we downsized the scope of the analysis and took into account those with FPKM (Fragments per kilobase of transcript per million fragments mapped) >100 and an absolute value of the Log2 ratio (amyR12/CICC2462) >1.0 and <−1.0. The numbers of genes were then markedly decreased to less than 350. For CICC2462-VS-amyR12, 290 unigenes presented marked expression differences, and 243 and 47 unigenes were up- and down-regulated in amyR12, respectively.

**Table 4:** Statistics of RNA-sequencing in *A. niger*

Sample name	*A*. *niger* CICC2462	amyR12
Clean reads	49,072,138	48,989,116
Genome map rate (%)	92.38	91.37
Gene map rate (%)	55.98	53.45
Expressed gene (%)	11,699	11646
Unique match genome (%)	89.19	87.93
Unique match gene (%)	54.31	53.36

### Important down-regulated genes in amyR12

As expected, the transcriptome data showed that the transcription levels of amylolytic enzyme, glucoamylase, glucosidase and amylase were markedly decreased in amyR12 (see Table 5). This finding is consistent with the above-presented results, which showed that the number of high secretory protein was significantly decreased. Along with a decrease in the total secreted protein, the level of proteases were also reduced. Moreover, some of the sugar transporter genes were also down-regulated. Similar changes have been found after the deletion of *xlnR* in *A. niger* [26]. However, the regulatory mechanism of sugar transporter remains unclear. We analysed the amyR-binding motif of the above-mentioned down-regulated genes by Blast searches. By screening the amyR-binding sequence in the promoter regions (−1 to −1000 with the putative translational start site as +1) of the genes, we found that the amylolytic enzyme genes contained at least one putative *amyR*-binding site in their promoter regions. The protease genes without the *amyR*-binding sequence were possible indirectly regulated by *amyR*, and some sugar transport genes have *amyR*-binding sequences (see Table 6).

**Table 5:** Transcript abundance of important down-regulated genes

Gene ID	CICC2462 -FPKM	amyR12 -FPKM	log2 ratio	FDR	Description
Amylolytic enzyme					
An03g06550	46,008.13	354.74	−7.02	0	Glucoamylase
An11g03340	11,537.46	42.07	−8.1	0	Acid-stable alpha-amylase
An12g06930	9907.17	55.93	−7.47	0	Alpha-amylase
An04g06920	3072.71	26.14	−6.89	0	Alpha-glucosidase
An01g10930	1630.37	14.7	−6.79	0	Alpha/beta-glucosidase agdC
Protease					
An06g00190	611.52	48.21	−3.67	0	Tripeptidyl-peptidase sed2
An06g00310	107.25	23.01	−2.22	8.85E−155	Carboxypeptidase S1
An01g00370	330.41	85.85	−1.94	1.78E−273	Aspartic endopeptidase (AP1)
An07g08030	110.6	3.27	−5.08	2.14E−285	Serine-type Carboxypeptidase F
An08g04490	215.81	17.92	−3.59	0	Endoprotease
Sugar transporter					
An15g03940	1891.82	158.73	−3.58	0	Glucose transporter rco-3
An12g07450	372.55	6.95	−5.74	0	Sugar transporter
An01g00850	117.57	2.28	−5.69	0	Sugar transporter
An02g00590	258.7	14.23	−4.18	0	Sugar transporter

**Table 6:** Positions of the possible *amyR* binding sites

CGGN8CGG			CGGN8AGG		Description
Gene ID	Coding strand	Non-coding strand	Coding strand	Non-coding strand	
An03g06550	−423 to −436	None	−192 to 205 −688 to −701	−320 to −333	Glucoamylase
An11g03340	None	None	None	None	Acid-stable alpha-amylase
An12g06930	−249 to −262	None	None	None	Alpha-amylase
An04g06920	−191 to −204 −574 to −587	None	None	None	Alpha-glucosidase
An01g10930	None	−904 to −917	-655 to -668	None	Alpha/beta-glucosidase agdC
An06g00190	None	None	None	None	Tripeptidyl-peptidase sed2
An06g00310	None	None	None	None	Carboxypeptidase S1
An01g00370	None	None	None	None	aspartic endopeptidase (AP1)
An07g08030	None	None	None	None	Serine-type carboxypeptidase F
An15g03940	−554 to −567	None	None	None	Glucose transporter rco-3
An12g07450	None	None	−702 to −715 −885 to −898	−867 to −880	Sugar transporter
An01g00850	None	None	−758 to −771	None	Sugar transporter
An02g00590	None	−857 to 870	−602 to 615	−950 to −963	Sugar transporter

The numbers represent the positions of the 5′ and 3′ ends of the amyR binding site with the putative translational start site as +1

**Fig. 2:**
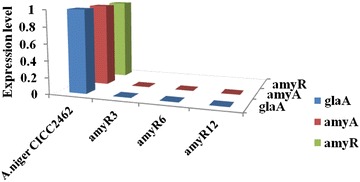
Quantitative real-time PCR of some representative transcripts that were differentially expressed in *A. niger* CICC2462 and ∆*amyR* (amyR3, amyR6 and amyR12) strains grown on fermentation medium. The selected genes were: glucoamylase gene *glaA*, amylase gene *amyA* and transcriptional activator *amyR*

To validate the reliability of the transcriptome analysis, quantitative real-time PCR analysis was performed on three differentially expressed genes, which were selected for their high expression levels (one transcription factor, *amyR*, and two highly expressed genes, *glaA* and *amyA*). The transcript abundance patterns of *A. niger* CICC2462 and amyR12 were compared with the RNA-Seq data. The results showed that for these three genes, real-time PCR revealed the same expression patterns as those detected in the RNA-Seq data (Fig. 2). The transcription levels of the *glaA* and *amyA* genes in the ∆*amyR* strain decreased to very low levels compared with the levels detected in the wild-type strain.

### Influence of the deletion of *amyR* on protease activity

**Fig. 3:**
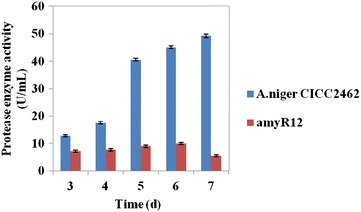
Activities of protease activities from *A. niger* CICC2462 and ∆*amyR* strains grown on fermentation medium

As mentioned above, the down-regulated genes in transcriptome data showed the transcription situation of protease genes. To analyze the mutant strain in more detail, protease activity analysis was carried out from these two strains (Fig. 3), compared with the wild-type strain, the protease activity was two- to tenfold lower in the amyR deletion strain.

### Up-regulation of genes in amyR12

Most of the oxidative phosphorylation and tricarboxylic acid cycle genes were up-regulated in *amyR12* (see Table 7). These include some genes relevant to oxidative phosphorylation, cytochrome C oxidase classes with heme-copper terminal oxidase activity (GO:0015002), ATP synthase classes and cytochrome C reductase classes with hydrogen ion transmembrane transporter activity (GO:0015078), NADH-ubiquinone oxidoreductase classes with NADH dehydrogenase (quinone) activity (GO:0050136), and succinate dehydrogenase (ubiquinone) class with succinate dehydrogenase activity (GO:0000104). These genes are mainly involved in the tricarboxylic acid cycle and include malate dehydrogenase (An07g02160), fumarate hydratase (An12g07850), succinate dehydrogenase (ubiquinone) flavoprotein subunit (An02g12770), and isocitrate dehydrogenase (NAD) subunit 2 (An08g05580). The transcriptome data showed that most of these genes related to these two central metabolic pathways are constitutively expressed. Normal cellular metabolism and the generation and utilization of energy were unaffected by deletion of the *amyR* gene in this organisms. In addition, ribosomal proteins (60S or 40S) with structural molecular activity (GO:0005198) that are mainly involved in the process of gene expression (GO:0010467) are concentrated in the up-regulated genes. Many unnamed protein products and hypothetical protein genes related to cytochrome and ribosomal proteins were found, although most have not been annotated, and some of these are available for novel gene prediction.

**Table 7:** Transcript abundance of genes involved in oxidative phosphorylation and the tricarboxylic acid cycle

Category/CBS513.88	CICC2462 -FPKM	amyR12 -FPKM	log2 ratio	Function
Oxidative phosphorylation				
An15g00690	100.27	222.14	1.15	NADH dehydrogenase (quinone)activity
An09g06850	129.19	273.96	1.08	NADH dehydrogenase (quinone)activity
An11g06200	188.04	397.72	1.08	NADH dehydrogenase (quinone)activity
An02g09730	157.37	369.6	1.23	NADH dehydrogenase (quinone)activity
An11g09390	159.65	340.95	1.09	NADH dehydrogenase (quinone)activity
An02g12770	389.46	891.41	1.19	Succinate dehydrogenase activity
An04g05220	162.43	450.23	1.47	Hydrogen ion transmembrane transporter activity
An08g06550	351.13	995.69	1.50	Hydrogen ion transmembrane transporter activity
An04g01200	337.24	844.5	1.32	Hydrogen ion transmembrane transporter activity
An09g06650	213.2	470	1.14	Hydrogen ion transmembrane transporter activity
An14g04080	318.99	696.8	1.13	Hydrogen ion transmembrane transporter activity
An11g10200	699.98	2394.31	1.77	Heme-copper terminal oxidase activity
An04g07180	212.62	714.57	1.75	Heme-copper terminal oxidase activity
An04g01560	425.74	1381.99	1.70	Heme-copper terminal oxidase activity
An14g04170	219.17	661.74	1.59	Heme-copper terminal oxidase activity
An02g01720	540.72	1611.1	1.58	Heme-copper terminal oxidase activity
An09g03990	107.19	263.52	1.30	Heme-copper terminal oxidase activity
An02g09930	402.47	936.69	1.22	Heme-copper terminal oxidase activity
An07g07390	525.54	1129.58	1.10	Heme-copper terminal oxidase activity
An12g04950	192.12	523.85	1.45	Hydrogen ion transmembrane transporter activity
An01g04930	194.67	462.54	1.25	Hydrogen ion transmembrane transporter activity;cation-transporting ATPase activity
An16g07410	659.64	1534.81	1.22	Hydrogen ion transmembrane transporter activity, cation-transporting ATPase activity
An14g04180	1366.84	3023.55	1.15	Hydrogen ion transmembrane transporter activity
An02g04520	195.69	399.15	1.03	Hydrogen ion transmembrane transporter activity
An01g04630	325.17	653	1.01	Hydrogen ion transmembrane transporter activity, cation-transporting ATPase activity
Tricarboxylic acid cycle				
An07g02160	282.02	924.35	1.71	Malate dehydrogenase activity
An12g07850	166.48	428.02	1.36	Hydro-lyase activity
An08g05580	386.02	899.66	1.22	Isocitrate dehydrogenase activity
An02g12770	389.46	891.41	1.19	Succinate dehydrogenase activity
An18g06760	594	1299.36	1.13	Isocitrate dehydrogenase activity
An14g04400	390.85	854.72	1.13	Succinate dehydrogenase activity

### Influence of the deletion of *amyR* on mycelium morphology

**Fig. 4:**
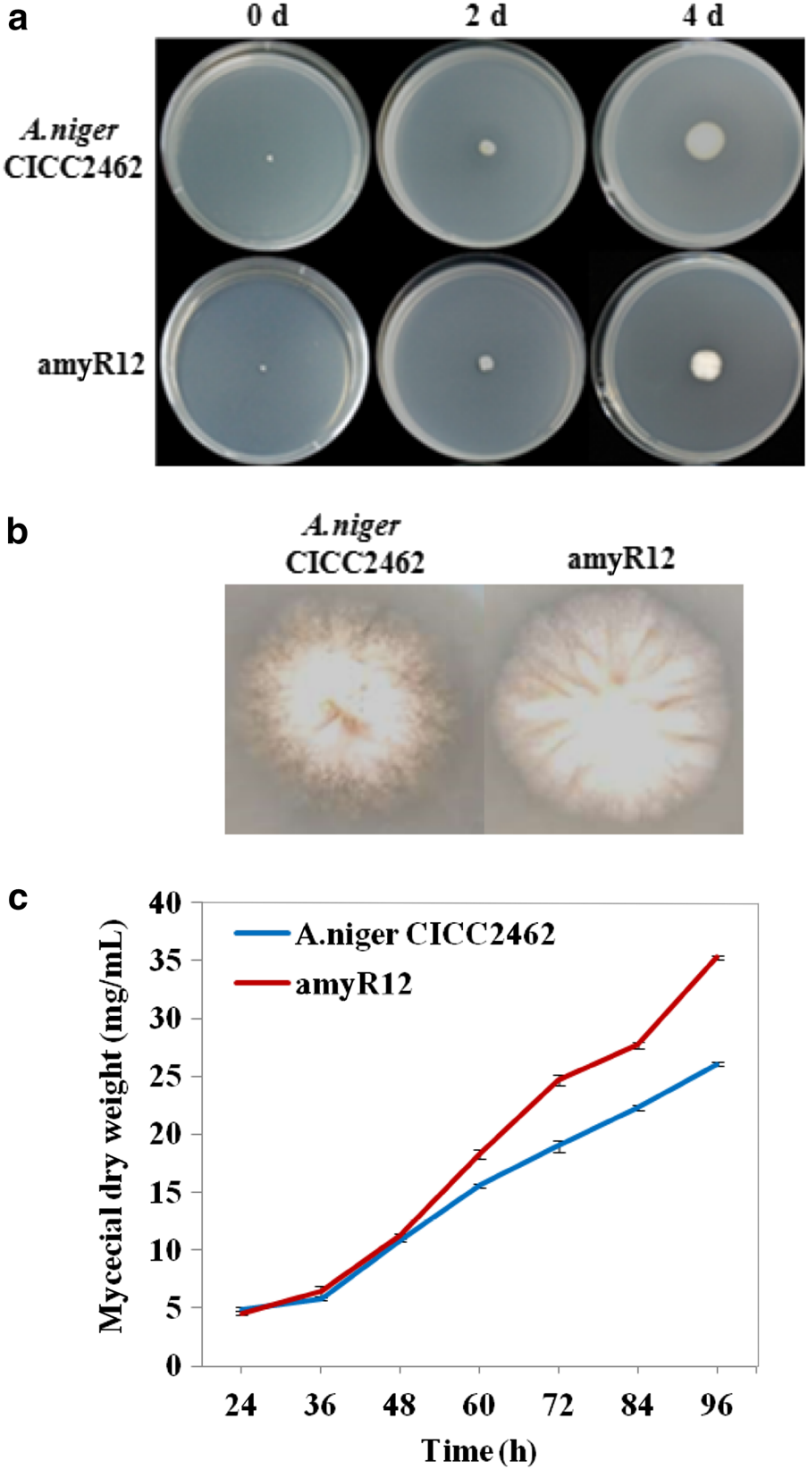
**a** Growth of *A. niger* CICC2462 and ∆*amyR* strains on PDA medium. **b** Growth of *A. niger* CICC2462 and ∆*amyR* strains on PDA medium after 8 days. **c** Biomass weights of *A. niger* CICC2462 and ∆*amyR* grown in fermentation medium

Up-regulation of the oxidative phosphorylation and tricarboxylic acid cycle genes is a good indication of increase in growth rate. The *A. niger* CICC2462 and *amyR12* strains were grown in PDA medium and the shake flask fermentation medium with glucose as the carbon source. Compared with the reference strain, *amyR12* on PDA medium did not differ in size and colour for the first few days (Fig. 4a), but a significant difference was observed after 8 days incubation in that the colony of the amyR12 strain is larger (Fig. 4b). In the shake flask fermentation medium, the mycelium of *amyR12* were thicker than those of the wild-type strain, and the dry weight of the *amyR12* strain increased higher growth rate than the wild type (Fig. 4c).

### Future perspectives

**Fig. 5:**
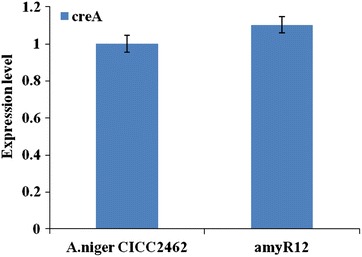
Quantitative real-time PCR of creA gene that was differentially expressed in *A. niger* CICC2462 and amyR12 strains grown on fermentation medium

The research and development of *filamentous fungus* expression systems has become a hotspot in the field of recombinant protein expression throughout the world. Previous studies have demonstrated that *Aspergillus* has great economic potential as an expression host for the secretion of proteins with industrial applications [4, 30, 31]. *Aspergillus niger* and *A. oryzae* are the most commonly used *Aspergillus* species for industrial production, and the host is crucial for the efficient expression of proteins [32]. The exhibited morphology influences not only the culture broth viscosity but also the productivity, substrate diffusion and downstream processing [33]. *Aspergillus niger* CICC2462 was selected since it is used in the industrial production of glucoamylase. This strain is a morphological mutant strain of *A. niger* that does not produce spores, has short mycelia, thick hyphae, which results in a low-viscosity fermentation broth, is a strong enzyme producer, has low protease activity, is osmotolerant, and is suitable for high-density submerged liquid fermentation. As described previously, glucose generally represses the expression of amylolytic genes in creA-dependent manner [34]. To determine the function of the *creA* gene, transcriptome data (CICC2462-FPKM = 117.34, amyR12-FPKM = 146.9 and log2 Ratio = 0.3242) and quantitative real-time PCR analysis (Fig. 5) of *creA* gene were performed in the two strains. Transcription is not different comparing *A. niger* CICC2462 and the amyR12 strains. Moreover, amylolytic enzyme activity in the creA deletion strain was detected in the presence of glucose as the carbon source. The enzyme activity of *A. niger* CICC2462 strain and creA deletion strain was 48966.0960 and 49121.0520 U/mL, respectively. The results show that there is only a small difference. The activity of the *creA* gene product is probably weaker in *A. niger* CICC2462. In the shake flask fermentation experiment, glucose still remain at a concentration of 3.8 % (*A. niger* CICC2462) and 4.5 % (∆*amyR*) in the medium after 4 days. There was no significant difference in consumption of glucose comparing the wild type and amyR deleted strains. The *A.niger* CICC2462 strain can also secrete a large number of enzyme proteins into the growth medium in the presence of a high concentration of glucose which was used as the sole carbon source. The regulation of the secretome is obviously different from other strains, indicating the great research value of this strain.

*amyR* has been studied in detail in several *Aspergillus* species in relation to amylolytic enzyme genes. The enzyme activity, SDS–PAGE and -omics analyses performed in our study provide clear indications for a key regulation role of *amyR*, because we observed strongly decreased levels of glucoamylase, amylase and glucosidase in our *amyR*-deletion strain compared with the wild type strain. We observed a reduction in protease secretion, possibly because the production of highly secreted proteins (glucoamylase, amylase and glucosidase) in amyR12 was decreased to a very low level. Considering the morphology, the amyR12 strain without growth inhibition compared to the wild type strain, and the dry weight of the amyR12 strain increased higher growth rate than the wild type. This result may be due to the reduced of highly expressed proteins in the liquid fermentation medium, thus nutrients are essentially used for mycelium growth.

Furthermore, promoter is a crucial element for controlling protein production and can largely regulate gene expression at the transcriptional level. The commonly used P*glaA* and P*amyA* promoters reduced their function in the ∆*amyR* strain. In general, homologous strong promoters appear to result in higher expression yields compared with heterologous promoters [31, 35]. As such, we need to determine the strong promoters in the ∆*amyR* strain. We primarily chose genes with a FPKM that was relatively high from the ∆*amyR* transcriptome data (see Table 8). The design and functional verification of promoters are a future line of research.

**Table 8:** Transcriptional levels of some genes in the *∆amyR* strain

Gene ID	FPKM	FDR	Description
An18g04840	6672.67	0	Elongation factor 1-alpha
An14g07060	6607.56	2.19E−07	Nitroreductase family protein
An03g06410	6318.12	0	C-4 methylsterol oxidase
An04g06510	5952.65	5.22E−121	Polyubiquitin
An08g04880	5706.63	0	Hypoxia induced family protein
An18g04220	5123.75	0	ADP/ATP carrier protein
An18g06650	5094.63	5.13E−87	Heat shock protein

## Conclusion

In this study, the regulatory effect of *amyR* on amylolytic enzymes in the expression system is apparent; specifically, the transcription and translation levels of glucoamylase and amylase were obviously decreased in the expression system. We now have a host strain with a low background of protein secretion and without growth inhibition, our work lays a foundation for continued improvements this expression system.
